# A Single Point Mutation within the Coding Sequence of Cholera Toxin B Subunit Will Increase Its Expression Yield

**DOI:** 10.6091/ibj.1165.2014

**Published:** 2014-07

**Authors:** Bita Bakhshi, Mina Boustanshenas, Masoud Ghorbani

**Affiliations:** 1 *Dept. of Bacteriology, Faculty of Medical Sciences, Tarbiat Modares University, Tehran, Iran; *; 2 *Dept. of Biology, Faculty of Basic Science, Science and Research Branch, Islamic Azad University, Tehran, Iran; *; 3 *Research and Production Complex, Pasteur Institute of Iran, Tehran, Iran*

**Keywords:** *Escherichia coli*, Point mutation, Cholera toxin B subunit (CTB), Protein expression

## Abstract

**Background: **Cholera toxin B subunit (CTB) has been extensively considered as an immunogenic and adjuvant protein, but its yield of expression is not satisfactory in many studies. The aim of this study was to compare the expression of native and mutant recombinant CTB (rCTB) in pQE vector.** Methods: ***ctx*B fragment from *Vibrio cholerae* O_1_ ATCC14035 containing the substitution of mutant *ctx*B for amino acid S128T was amplified by PCR and cloned in pGETM-T easy vector. It was then transformed to *E. coli* Top 10F' and cultured on LB agar plate containing ampicillin. Sequence analysis confirmed the mature *ctx*B gene sequence and the mutant one in both constructs which were further subcloned to pQE-30 vector. Both constructs were subsequently transformed to *E. coli* M15 (pREP4) for expression of mature and mutant rCTB. **Results: **SDS-PAGE analysis showed the maximum expression of rCTB in both systems at 5 hours after induction and Western-blot analysis confirmed the presence of rCTB in blotting membranes. The expression of mutant rCTB was much higher than mature rCTB, which may be the result of serine-to-threonine substitution at position 128 of mature rCTB amino acid sequence created by PCR mutagenesis. The mutant rCTB retained pentameric stability and its ability to bind to anti- cholera toxin IgG antibodies. **Conclusion:** Point mutation in *ctx*B sequence resulted in over-expression of rCTB, probably due to the increase of solubility of produced rCTB. Consequently, this expression system can be used to produce rCTB in high yield.

## INTRODUCTION

Cholera is a severe diarrheal disease which still remains as an epidemic or endemic threat in many parts of the world, particularly in developing countries [1]. *Vibrio cholerae* is classified into more than 200 serotypes on the basis of somatic antigens [2]. Seven cholera epidemics which are caused by only O1 and O139 serogroups have occurred, and its outbreaks continue to occur in Iran and other developing countries [3-5]. Cholera is mainly caused in countries with poor sanitations, and many attempts have been performed to develop an improved vaccine [6]. 

Cholera symptoms are mainly caused by cholera toxin [7], an 85-kDa protein. This protein is composed of two subunits: a single A subunit (cholera toxin A [CTA]) which is responsible for activation of adenylate cyclase in the intestinal cells, and B subunit (cholera toxin B [CTB], 11.6 kDa), which binds the respective holotoxin to its intestinal receptor (ganglioside GM1 [monosialotetrahexosylganglioside] [8, 9]. 

CTB has recently attracted many interests as an adjuvant for various other peptide or carbohydrate antigens [10, 11]. It comprises a transmucosal carrier delivery system for induction of oral tolerance when conjugated to antigens and allergens [7, 12]. When CTB is chemically or genetically conjugated to poor immunogenes, it can elicit serum and secretory antibodies against the fused antigens [13, 14]. Vaccination against cholera is a powerful prevention strategy, because it can provide long-term protective immunity [15-17]. Based on this realization, a variety of vaccines against cholera were developed that divided into two principal kinds, the killed and live attenuated *Vibrio cholerae* vaccines [6]. Various hosts have been used to develop a high-level expression system for producing recombinant CTB (rCTB), but most of these attempts were failed. The aim of this study was to evaluate two different strategies for expression of rCTB in pQE-30 vector and to compare the level of native and mutant *ctx*B gene expression using PCR mutagenesis.

## MATERIALS AND METHODS

***Isolation and cloning of ctxB gene.*** Specific primers were designed according to *ctx*B sequence of *V. cholerae* O1 ATCC14035 obtained from NCBI and cutting sites of *Bsp*HI and *Xho*I were designed within forward and reverse primer sequences, respectively. Thrombin sequence was designed within the 5' terminal of reverse primer. PCR was performed using *ctx*B-F (5'GCG TCA TGA TTA AAT TAA AAT TTG GTG TTT TTT TTA CAG TTT TAC3')/*ctx*B-R (5'CGC CTC GAG GGA ACC GCG TGG CAC CAG ATT TGC CAT AGT AATTG 3') primers with the following program: denaturation step at 94°C for 5 min, 25 cycles at 94°C for 45 s, 58°C for 45 s, and 72°C for 1 min (using genomic DNA of* V. cholerae* O1 ATCC14035 as DNA template). After amplification, the *ctx*B fragment was electrophoresed on 1% (wv^-1^) agarose gel (Fermentas, Burlington, Canada) and then purified and cloned in the pGEM-T easy vector (Promega, Canada). The construct was then transformed to *Escherichia coli* Top 10F' (Invitrogen, Carlsbad, CA). White colonies on LB agar plate (100 µg l^-1^ ampicillin, 40 µg l^-1^ IPTG, and 30 µg ^-l^ X-gal) were selected and used for plasmid extraction using QIAprep Spin Miniprep Kit (Qiagen, Valencia, CA, USA). The *ctx*B sequence was confirmed by restriction fragment length polymorphism and sequence analysis (Genfanavaran, Macrogen, Seoul, Korea) of extracted plasmids with SP6 and T7-promoter universal primers. The pGEM-T_*ctx*B construct was digested with *Bsp*HI and *Xho*I, and *ctx*B was subcloned to *Xho*I and *Nco*I digested pQE-30 (Qiagen, Canada). The construct was transformed to competent *E. coli* M15 (pREP4) (Qiagen, Canada).

***Expression of recombinant*** ***cholera toxin B subunit*** ***.*** Transformed *E. coli* M15 (pREP4) cells (Qiagen, Canada) harboring pQE-*ctx*B construct were grown in LB medium containing ampicillin (100 µg l^-1^) and incubated at 37°C with shaking (200 rpm) until optimal density at 600 nm reached to 0.6-0.8. The IPTG (Sigma-Aldrich, Germany) was added to the culture at a final concentration of 1 mM. Additional 5-h incubation with shaking was carried out, and culture sampling was performed in each hour. Bacterial cells were harvested by centrifugation at 4000 ×g at 4°C for 20 min. 

***SDS-PAGE analysis and Western-blotting.*** The pellet of bacterial cells was lysed by 200 µl lysis buffer (25 mM TRIS-Cl and 2 mM EDTA, pH 7.6) for each sample following sonication. Lysed pellets were mixed with sample buffer, boiled for 10 min and electrophoresed on two separate SDS-PAGE gels (15% wv^-1^) under the same running conditions. One gel was stained with Coomassie Brilliant Blue R-250 (1% wv^-1^), and the other one was subjected to blotting onto poly (vinylidene difluoride) membrane (Hi-bond Amersham Biosciences, Piscataway, NJ, USA). Western-blot analysis was performed using 1:1000 dilution of rabbit polyclonal anti-cholera toxin antibody (Sigma-Aldrich, Germany) in PBS and 1:10000 dilution of horseradish peroxidase-conjugated goat anti-rabbit IgG (Sigma-Aldrich, Germany) in PBS as primary and secondary antibodies, respectively. The production of rCTB was detected with bound antibodies using a chemiluminescent substrate, electrophoresis chemiluminescent (Hi-bond Amersham Biosciences, Piscataway, NJ, USA) and then exposed to Kodak X-OMAT Blue Autoradiography Film. The amount of expressed rCTB was evaluated using FireReader D56 software (UVI Tec, UK). 

***Over-expression of mutant recombinant*** ***cholera toxin B subunit*** ***.*** Mutant rCTB gene was design via replacing the 383^th^ nucleotide of mature *ctx*B gene (guanine instead of cytosine). PCR reaction was carried out using *ctx*B-F (5'GCG TCA TGA TTA AAT TAA AAT TTG GTG TTT TTT TTA CAG TTT TAC3')/*ctx*B-R (5'CGC CTC GAG GGA ACC GCG TGG CAC CAG ATT TGC CAT A**G**T AATTG 3') primers. The base in bold in reverse primer sequence is the substitution base. Mutant *ctx*B fragment was cloned in pGEM and subcloned in pQE-30 for expression the mutant rCTB as described previously. Expression of this new mutant rCTB was analyzed with SDS-PAGE gel (15% wv^-1^) and confirmed by Western-blot analysis. The amount of produced mutant rCTB was evaluated using FireReader D56 software (UVI Tec, UK).

## RESULTS

**Fig. 1:**
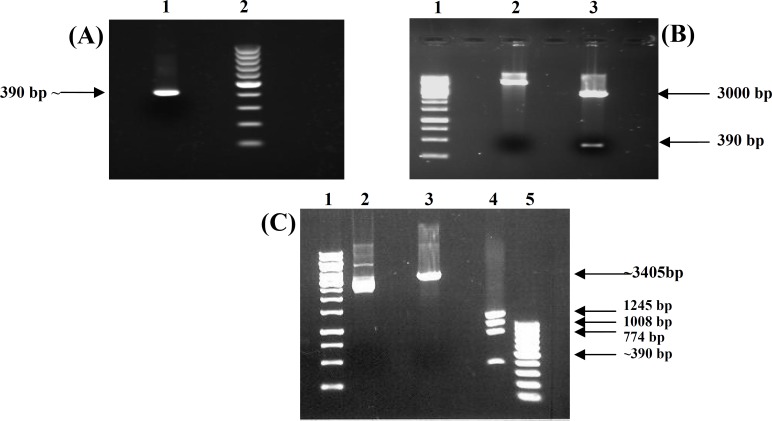
Agarose gel electrophoresis for detection of *amplified* DNA bands. **A)** Purification of amplified *ctx*B fragment from agarose gel. Lane 1, *ctxB *gene amplified w**i**th PCR from *Vibrio cholerae* and purified from agarose gel and lane 2, 100 bp DNA marker. **B) **Digested pGEM-T-*ctx*B plasmid with *Eco*RI restriction enzyme. Lane 1, 1 kb DNA size marker; lane 2, undigested pGEM-T containing *ctx*B fragment and lane 3, digested pGEM-T-*ctx*B plasmid with *Eco*RI restriction enzyme. **C) **pGEM-T-*ctx*B plasmid. Lane 1, 1 kb DNA size marker; lane 2, undigested pGEM-T-*ctx*B; lane 3, digested pGEM-T-*ctx*B with *Xho*I; lane 4, double digested pGEM-T-*ctx*B with *Xho*I and *Bsp*HI, single 390 bp band corresponded to *ctx*B gene and lane 5, 100 bp DNA marker.

***Amplification and cloning of ctxB.*** The expected PCR product, a single band of 390 bp for *ctx*B gene, was obtained (Fig. 1A). The recombinant pGEM-T-*ctx*B plasmid extracted from *E. coli* Top10 was digested with *EcoR*I. Two bands of 3,000 bp and 390 bp were obtained related to pGEM-T vector and *ctx*B gene, respectively (Fig. 1B). The pGEM-T-*ctx*B was double digested with *Bsp*HI and *Xho*I restriction enzymes; approximately a 390-bp band corresponding to *ctx*B was appeared in agarose gel (Fig. 1C). Finally, the *ctx*B sequences in pGEM-T-*ctx*B were confirmed by DNA sequencing.

***Construction of mutant ctxB sequence.*** Mutant *ctx*B gene was produced using designed primers with a point mutation in reverse primer sequence, which belonged to 383^th^ nucleotide of mature *ctx*B gene (cytosine was replaced with guanine in mutant *ctx*B gene). This point mutation finally produced threonine amino acid instead of serine in mutant rCTB protein. Both of these amino acids had polar, uncharged R group in their chemical structure (CH_2_OH for serine and CHOHCH_3_ for threonine). The sequence of mutant *ctx*B was confirmed by DNA sequence analysis. 

**Fig. 2:**
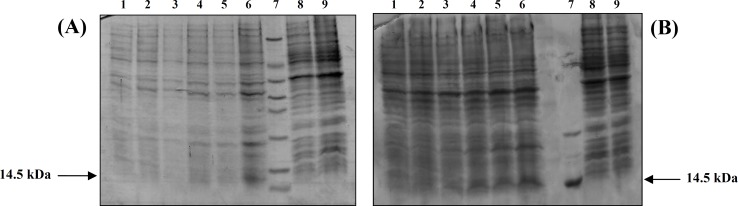
Comparison of SDS-PAGE analysis of mature and mutated recombinant CTB in *E. coli*. **A)** SDS-PAGE analysis of expression of mature recombinant CTB in *E. coli* M15 (pREP4) using pQE_*ctx*B as expression vector. Lane 1, M15 (pREP4) containing pQE_*ctx*B before induction with IPTG; lane 2-6, indicate 1-5 hours after induction respectively; lane 7, protein size marker and lane 8 and 9, M15 (pREP4) containing pQE used as negative control.** B**) SDS-PAGE analysis of expression of mutant recombinant CTB in *E. coli* M15 (pREP4using pQE containing mutant *ctx*B gene as expression vector. Lane 1, M15 (pREP4) containing pQE_*ctx*B before induction with IPTG; lane 2-6, indicate 1-5 hours after induction respectively; lane 7, commercial CT (cholera toxin) used as positive control and lane 8 and 9, M15 (pREP4) containing pQE used as negative control

**Fig. 3:**
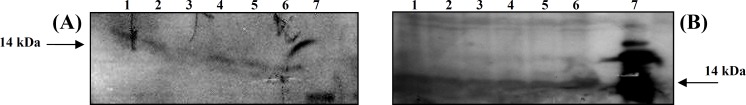
Western-blot analysis of mature and mutated recombinant CTB. **A)** Western-blot analysis of heated samples containing mature recombinant CTB probed with anti-CT antibody. Lane 1, expression of rCTB in M15 (pREP4) containing pQE-*ctx*B plasmid before induction with IPTG; lane 2-6, indicate 1-5 hours after induction respectively and lane 7, commercial CT used as positive control.** B)** Western-blot analysis of heated samples containing mutant recombinant CTB probed with anti-CT antibody. Lane 1, expression of rCTB in M15 (pREP4) containing pQE-*ctx*B plasmid before induction with IPTG; lane 2-6, indicate 1-5 hours after induction respectively and lane 7, commercial CT used as positive control

***Expression of mature and mutant*** ***recombinant cholera toxin B subunit*** ***.*** Two constructs of pQE vector, one containing native *ctx*B fragment and the other containing mutant *ctx*B gene were transformed separately into *E. coli* M15 (pREP4). Time gradient was applied to recognize the best time for producing maximum amount of rCTB in both constructs. The best condition to produce rCTB in both constructs was determined at 37°C, 1 mM concentration of IPTG and 5 hours after induction (Fig. 2A and 2B). A major band of approximately 14.5 kDa corresponding to rCTB was observed on SDS-PAGE analysis of both constructs. Comparison of SDS-PAGE analysis of the two different constructs using FireReader D56 software (UVI Tec, UK) demonstrated that the expression of mutant rCTB was assessed to be approximately 80 mg l^-1^, while the expression of mature rCTB was about 7.8 mg l^-1^ (approximately 10 fold higher than mature rCTB in the same conditions) (Fig. 2A and 2B). Over-expression of mutant rCTB may be occurred due to replacement of serine for threonine in CTB protein structure via point mutation. Antigenicity of expressed proteins was confirmed by immunoblotting using anti- cholera toxin IgG. The over-expression of mutant rCTB in contrast with mature rCTB was observed after exposure protein bands on a blue autoradiography film (Fig. 3A and 3B).

## DISCUSSION

CTB has been extensively studied as an immunogen or adjuvant in intestinal and nasal mucosal sites [15, 18]. It has ability to deliver covalently attached antigens to the mucosal cells via binding to GM_1_ ganglioside receptor on the surface of epithelial cells [19, 20]. CTB was used as an adjuvant to increase the immune response level in many different vaccines specially vaccines against toxigenic *Vibrio cholerae* strains [21-23]. Many efforts have been performed to produce CTB in *E. coli* [24], *Lactobacillus* and *Bacillus brevis* [25, 26] or even *Vibrio cholerae* strains lacking the CTA gene [7]. Yeasts (*Saccharomyces cerevisiae*) and plants (such as tomato, potato, and tobacco) have been also used to produce rCTB, but the amount of expressed protein is too low in former systems [27]. The sequence analysis of cholera toxin revealed a number of mutations at different positions on the genome sequence of both cholera toxin subunits across different serogroups. Some of these mutations are non-synonymous such as polar to non-polar or vice-versa. However, some other, especially at structurally related positions, may be important for understanding the pathogenesis of different serogroups or for improvement of recombinant cholera toxin proteins to produce vaccines against multiple serogroups [28]. The substitution of H18Y for T47I in CTB of El Tor strains was reported by Ansaruzzaman and colleagues [29]. Kumar *et al.* [30] reported a novel mutation from India at position 20 of CTB sequence (H20N) of the *Vibrio cholerae *O1 El Tor biotype [30].

In the present study, rCTB was expressed in *E. coli* M15 (pREP4) using pQE-30 vector. The sequence of *ctx*B gene was manipulated by a point mutation on 383^th^ nucleotide (guanine instead of cytosine) and consequently by serine-to-threonine substitution in mature CTB amino acids sequence at position 128. Moreover, the mutant retained pentameric stability and high affinity binding to anti- cholera toxin IgG used in Western-blot analysis. This nucleotide substitution was chosen because the subsequent substitution occurs between two amino acids which are in the same group with polar, uncharged R group in their chemical structure. Some properties of these two amino acids such as molecular weight and solubility are similar, therefore the expression of mutant rCTB without changing in conformation or biological properties could be guaranteed. It was illustrated that point mutation (serine-to-threonine substitution at position 128 on amino acid sequence of rCTB) caused over-expression of mutant rCTB (approximately 10 fold higher than mature rCTB). In addition, the expressed mutant rCTB retained pentameric stability and affinity to bind to anti- cholera toxin IgG. The over-expression of mutant rCTB in the present study may be probably due to the increase in the solubility of mutant protein via serine-to-threonine substitution and decrease in the amount of protein expressed as inclusion bodies. 

Some different studies have investigated the effects of different mutations or manipulations of the CTB sequence. Merrite and colleagues [31] reported a point mutation at amino acid Gly(33) of the CTB sequence (Gly33Arg), which led to increase in the affinity of CTB to bind to receptors. In the study by Silva *et al.* [32], the substitution of G for A residue was performed by site-directed mutagenesis at 182^th^ nucleotide of native CTB sequence, which ultimately resulted in the production of glutamate instead of glycine. This process abolished the biding of CTB to GM1 without affecting the expression level of CTB protein.

Arêas and colleagues [19] were designed a native* ctx*B protein sequence according to the codon usage of *E. coli*, Lactobacillus* casei*, and *Salmonella typhimurium*. They manipulated the *ctx*B coding sequence by substitution of some nucleotides for the purpose of an over-expression of CTB protein in pAE-*ctx*B system [19]. 

Aman and colleagues [33] reported the engineering and crystallographic structure of a mutant cholera toxin, with histidine-to-alanine substitution at position 57 in the B subunit. Although the mutant protein retained its pentameric stability and high affinity binding to GM1 ganglioside, its immunomodulatory activity was lost due to this substitution [33].

Our previously published data have indicated the pQE-*ctx*B construct as a highly efficient system to produce rCTB [34] and high affinity binding to GM1 ganglioside [35]. Some advantages of this construct are as follow: i) pQE30 contains the T5 promoter that is recognized by the *E. coli* host RNA polymerase. This promoter is under the control of *lac* operon, which can be induced by IPTG and is one of the powerful promoters to express target genes. ii) The recombinant proteins, which were expressed with extra 6xHis-tag sequence at C-terminus of proteins in pQE vector, enable us to use Ni^+2^-charged column chromatography to purify recombinant proteins. iii) The extra thrombin sequence, which was added to C-terminus of rCTB, can facilitate the separation of His-tag sequence from expressed protein.

In conclusion, pQE plasmid can express a high level of rCTB in *E. coli* M15 (pREP4) when S128T amino acid substitution was performed by PCR mutagenesis. *E. coli* still remains one of the most useful organisms to produce recombinant proteins compared to other bacteria, and this expression system can be used to produce rCTB in high yield with particular manipulations. 

## References

[1] Albert MJ, Siddique AK, Islam MS, Faruque AS, Ansaruzzaman M, Faruque SM (1993 Mar). Large outbreak of clinical cholera due to Vibrio cholerae non-O1 in Bangladesh. Lancet.

[2] Faruque SM, Albert MJ, Mekalanons JJ (1998 Dec). Epidemiology, genetics, and ecology of toxigenic Vibrio cholerae. Microbiol Mol Biol Rev.

[3] Kanungo S, Sah BK, Lopez AL, Sung JS, Paisley AM, Sur D (2010 Mar). Cholera in India: an analysis of reports, 1997–2006. Bull World Health Organ.

[4] Bakhshi B, Pourshafie MR (2009). Assessing clonality of Vibrio cholerae strains isolated during four consecutive years (2004-2007) in Iran. Scand J Infect Dis.

[5] Bakhshi B, Pourshafie MR, Navabakbar F, Tavakoli A, Shahcheraghi F, Salehi M (2008 Jul). Comparison of distribution of virulence determinants in clinical and environmental isolates of Vibrio cholerae. Iran Biomed J.

[6] Thungapathra M, Sharma C, Gupta N, Ghosh RK, Mukhopadhyay A, Koley H (1999 Jun). Construction of a recombinant live oral vaccine from a non-toxigenic strain of Vibrio cholerae O1 serotype inaba biotype E1 Tor and assessment of its reactogenicity and immunogenicity in the rabbit model. Immunol Lett.

[7] Rudin A, Riise GC, Holmgren J (1999 Jun). Antibody responses in the lower respiratory tract and male urogenital tract in humans after nasal and oral vaccination with cholera toxin B subunit. Infect Immun.

[8] Holmgren J (1981 Jul). Actions of cholera toxin and the prevention and treatment of cholera. Nature.

[9] Holmgren J, Fredman P, Lindblad M, Svennerholm AM, Svennerholm L (1982 Nov). Rabbit intestinal glycoprotein receptor for Escherichia coli heat-labile enterotoxin lacking affinity for cholera toxin. Infect Immun.

[10] de Aizpurua HJ, Russell-Jones GJ (J Exp Med.1988 Feb). Oral vaccination. Identification of classes of proteins that provoke an immune response upon oral feeding.

[11] Glass RI, Holmgren J, Khan MR, Hossain KM, Huq MI, Greenough WB (1984 Apr). A randomized, controlled trial of the toxin-blocking effects of B subunit in family members of patients with cholera. J Infect Dis.

[12] Li TK, Fox BS (1996 Dec). Cholera toxin B subunit binding to an antigen presenting cell directly co-stimulates cytokine Expression from a T cell clone. Int Immunol.

[13] Gong Z, Long X, Pan L, Le Y, Liu Q, Wang S (2009 Aug). Cloning, expression, purification and characterization of the cholera toxin B subunit and triple glutamic acid decarboxylase epitopes fusion protein in Escherichia coli. Protein Expr Purif.

[14] Harakuni T, Sugawa H, Komesu A, Tadano M, Arakawa T (2005 Sep). Heteropentameric cholera toxin B subunit chimeric molecules genetically fused to a vaccine antigen induces systemic and mucosal immune responses: a potential new strategy to target recombinant vaccine antigens to mucosal immune systems. Infect Immun.

[15] Miyata T, Harakuni T, Tsuboi T, Sattabongkot J, Kohama H, Tachibana M (2010 Sep). Plasmodium vivax ookinete surface protein Pvs25 linked to cholera toxin B subunit induces potent transmission-blocking immunity by intranasal as well as subcutaneous immunization. Infect Immun.

[16] Kenner JR, Coster TS, Taylor DN, Trofa AF, Barrera-Oro M, Hyman T, Adams JM, Beattie DT, Killeen KP, Spriggs DR (1995 Oct). Peru-15, an improved live attenuated oral vaccine candidate for Vibrio cholerae O1. J Infect Dis.

[17] Taylor DN, Killeen KP, Hack DC, Kenner JR, Coster TS, Beattie DT (1994 Dec). Development of a live, oral, attenuated vaccine against El Tor cholera. J Infect Dis.

[18] Bergquist C, Johansson EL, Lagergard T, Holmgren J, Rudin A (1997 Jul). Intranasal vaccination of humans with recombinant cholera toxin B subunit induces systemic and local antibody responses in the upper respiratory tract and the vagina. Infect Immun.

[19] Arêas APM, Oliveira MLS, Ramos CRR, Sbrogio-Almeida ME, Raw I, Ho PL (2002 Aug). Synthesis of cholera toxin B subunit gene: cloning and expression of a functional 6XHis-tagged protein in Escherichia coli. Protein Expr Purif.

[20] Bergerot I, Ploix C, Petersen J, Moulin V, Rask C, Fabien N (1997 Apr). A cholera toxoid-insulin conjugate as an oral vaccine against 486 spontaneous autoimmune diabetes. Proc Natl Acad Sci USA.

[21] Fontana MR, Monaci E, Yanqing L, Guoming Q, Duan G, Rappuoli R (2000 Aug). IEM101, a naturally attenuated Vibrio cholerae strain, as carrier for genetically detoxified derivatives of cholera toxin. Vaccine.

[22] Ryan ET, Calderwood SB () (2000 Aug). Cholera vaccines. Clin Infect Dis.

[23] Rijpkema SGT, Bik EM, Jansen WH, Gielen H, Versluis LF, Stouthamer AH (1992 Jun). Construction and analysis of a Vibrio cholerae delta-aminolevulinic acid auxotroph which confers protective immunity in a rabbit model. Infect Immun.

[24] L'hoir C, Renard A, Martial JA (1990 Apr). Expression in Escherichia coli of two mutated encoding the cholera toxin B subunit. Gene.

[25] Slos P, Dutot P, Reymund J, Kleinpeter P, Prozzi D, Kieny MP (1998 Dec). Production of cholera toxin B subunit in Lactobacillus. FEMS Microbiol Lett.

[26] Goto N, Maeyama J, Yasuda Y, Isaka M, Matano K, Kozuka S (2000 Apr). Safety evaluation of recombinant cholera toxin B subunit produced by Bacillus brevis as a mucosal adjuvant. Vaccine.

[27] Arzanlou M, Rezaee A, Shahrokhi N, Hossini A, Yasuda Y, Tochikubo K (2005). Expression of cholera toxin B subunit in Saccharomyces cerevisiae. Ann Microbiol.

[28] Shamini G, Ravichandran M, Sinnott JT, Somboonwit C, Sidhu HS, Shapshak P (2011 Mar). Structural inferences for Cholera toxin mutations in Vibrio cholerae. Bioinformation.

[29] Ansaruzzaman M, Bhuiyan NA, Nair BG, Sack DA, Lucas M, Deen JL (2004 Nov). Cholera in Mozambique, variant of Vibrio cholera. Emerg Infect Dis.

[30] Kumar P, Jain M, Goel AK, Bhadauria S, Sharma SK, Kamboj DV (2009 Feb). A large cholera outbreak due to a new cholera toxin variant of the Vibrio cholerae O1 El Tor biotype in Orissa, Eastern India. J Med Microbiol.

[31] Merritt EA, Sarfaty S, Jobling MG, Chang T, Holmes RK, Hirst TR (1997 Jul). Structural studies of receptor binding by cholera toxin mutants. Protein Sci.

[32] Silva A, Fando R, Benitez JA (1998 Oct). Overexpression of a mutant B subunit in toxigenic Vibrio cholerae diminishes production of active cholera toxin in vivo. Curr Microbiol.

[33] Aman AT, Fraser S, Merritt EA, Rodigherio C, Kenny M, Ahn M (2001 Jul). A mutant cholera toxin B subunit that binds GM1- ganglioside but lacks immunomodulatory or toxic activity. Proc Natl Acad Sci USA.

[34] Boustanshenas M, Bakhshi B, Ghorbani M, Norouzian D (2012). Comparison of two recombinant systems for expression of cholera toxin B subunit from Vibrio cholerae. Indian J Med Microbiol.

[35] Mina Boustanshenas, Bita Bakhshi, Masoud Ghorbani (2013 Feb). Investigation of immunological responses against a native recombinant CTB-whole cell Vibrio cholerae vaccine in a rabbit model. J Appl Microbiol.

